# Gram-positive Rod Surveillance for Early Anthrax Detection

**DOI:** 10.3201/eid1109.041013

**Published:** 2005-09

**Authors:** Elizabeth M. Begier, Nancy L. Barrett, Patricia A. Mshar, David G. Johnson, James L. Hadler, Connecticut Bioterrorism Field Epidemiology Response Team

**Affiliations:** * Connecticut Department of Public Health, Hartford, Connecticut, USA;; † Centers for Disease Control and Prevention, Atlanta, Georgia, USA

**Keywords:** population surveillance, gram-positive rods, anthrax, clostridium infections, sepsis, disease notification, dispatch

## Abstract

Connecticut established telephone-based gram-positive rod (GPR) reporting primarily to detect inhalational anthrax cases more quickly. From March to December 2003, annualized incidence of blood isolates was 21.3/100,000 persons; reports included 293 *Corynebacterium* spp., 193 *Bacillus* spp., 73 *Clostridium* spp., 26 *Lactobacillus* spp., and 49 other genera. Around-the-clock GPR reporting has described GPR epidemiology and enhanced rapid communication with clinical laboratories.

Identifying intentional *Bacillus anthracis* exposures quickly is essential for limiting human illness and death (*1*). During the 2001 anthrax attack, inhalational anthrax developed in 11 persons, and 5 died (*2*). Initial laboratory evidence of anthrax infection came from routine diagnostic blood cultures, which yielded *B*. *anthracis* in all 8 patients, who had not received antimicrobial drug therapy before blood cultures were obtained (*3**,**4*). Less than 24 hours elapsed from the time each patient's blood was drawn and the culture inoculated, until their culture was initially noted to have bacterial growth and preliminarily identified as gram-positive rods by immediate microscopic examination of a Gram stain. However, species-specific identification generally took several more days since additional laboratory testing of the bacterial isolate was required.

The Connecticut inhalational anthrax patient was intubated for mechanical ventilation during the 2-day delay between preliminary identification of gram-positive rods in blood culture and laboratory results specifically suggesting *B*. *anthracis*. According to then-existing requirements, the Connecticut Department of Public Health (CDPH) was not notified until *B*. *anthracis* was suspected. Public health officials were unable to interview the patient, who never recovered.

Since January 1, 2003, Connecticut laboratories and physicians have been required to report any gram-positive rod (GPR) identified from blood or cerebrospinal fluid (CSF) to CDPH. CDPH requested that laboratories call immediately if the isolate was identified within 32 hours of inoculation. This was the first time CDPH required laboratories to report a finding immediately by telephone. Surveillance objectives were to detect anthrax septicemia or meningitis more quickly, ensure around-the-clock laboratory reporting of potential bioterrorism events, and describe the epidemiology of GPR septicemia and meningitis in the absence of an intentional *B*. *anthracis* release.

Across the nation, local, state, and federal agencies have been pilot testing a variety of surveillance approaches to detect intentional disease outbreaks more quickly (*5**–**10*). Approaches have included syndromic surveillance (*6**–**8*) and environmental air monitoring for potential bioterrorism agents (*9**,**10*). We describe results from the inaugural year of CDPH's unique laboratory-based surveillance system.

## The Study

At the end of January 2003, Connecticut clinical laboratories were notified by mail that GPR isolates identified from CSF or blood within 72 hours of culture inoculation must be reported to CDPH Epidemiology Program. CDPH asked laboratories to call the department immediately if the isolate was identified within 32 hours of inoculation and collected either from an outpatient or an inpatient within 3 days of admission. Other GPR reports were to be mailed to CDPH. Although CDPH was most interested in timely telephone reporting of isolates identified within 24 hours of inoculation, we chose 32 hours to identify isolates missed in laboratories lacking sufficient staff to continuously examine blood cultures during night shifts (generally 8-hour periods). Blood cultures were processed according to each clinical laboratory's usual culture practices since reported culture isolates were obtained from routine diagnostic testing. In clinical settings, blood cultures are generally performed by filling commercially manufactured bottles, primed to promote either anaerobic or aerobic bacterial growth, with the patient's blood at the time of phlebotomy. Culture bottles are then brought to the clinical laboratory for incubation.

Immediate clinical follow-up was conducted whenever >1 of the patient's blood culture bottles yielded the isolate within 32 hours of inoculation and for all CSF isolates. This follow-up involved clinically characterizing the patient's illness through telephone discussion with the patient's physician or inpatient nurse to determine whether the illness was suspicious for anthrax (e.g., respiratory symptoms or widened mediastinum seen on chest radiograph). Laboratory follow-up was conducted for all isolates, with daily laboratory contact until genus identification. For *Bacillus* spp., laboratories were asked to report isolates' hemolysis and motility characteristics, and, if necessary, isolates were forwarded to Connecticut's state laboratory to rule out *B*. *anthracis* by γ-phage lysis.

Laboratory audits were conducted to ensure complete reporting of qualifying isolates; 33 of Connecticut's 34 clinical laboratories participated. We provided laboratories a list of GPR genera, and they provided a list of blood and CSF cultures that had yielded these genera within 72 hours of inoculation during 2003. We compared patient names, culture dates, and results with the 2003 GPR reports to identify unreported isolates.

Chart reviews were performed for *Clostridium* isolates to obtain etiology and underlying medical conditions. Health department labor resources were estimated by staff questionnaire administered October 2003. Because laboratories required several weeks to implement the reporting requirement after notification, the analysis period was limited to March–December 2003. In addition, only the first isolate from a given patient's illness was counted in this analysis.

From March to December 2003, a total of 623 GPR isolates were identified. CSF isolates were few (5 total: 2 *Listeria* spp., 2 *Bacillus* spp., and 1 *Corynebacterium* sp.). By genus, blood isolates included 293 *Corynebacterium* spp., 193 *Bacillus* spp. (none *B*. *anthracis*), 73 *Clostridium* spp., 26 *Lactobacillus* spp., 14 *Listeria* spp., 10 *Propionibacterium* spp., and 9 other genera (Table 1). Annualized incidence of GPR blood isolates was 21.3/100,000 persons. Twenty-three of the 195 *Bacillus* isolates were forwarded to Connecticut's state laboratory to rule out *B*. *anthracis* by γ-phage lysis (all were negative).

**Table 1:** Characteristics of gram-positive rod bacterial isolates from blood culture, Connecticut, March–December 2003

Genus	Total	Reported n* (%)	Time from inoculation to growth				No. inoculated bottles with growth by bottle type		
			No. isolates†	Median (h)	Range (h)	% positive <24 h	No. isolates†	No. aerobic inoculated (% aerobic positive)	No. anaerobic inoculated (% anaerobic positive)
*Bacillus‡*	193	134 (69)	161	23.5	2.7–70.3	56	134	242 (43)	218 (21)
*Clostridium*	73	47 (64)	69	15.3	1.4–71.9	75	70	134 (13)	134 (68)
*Corynebacterium‡*	293	94 (32)	220	42.9	2.8–71.9	8	94	178 (49)	174 (15)
*Lactobacillus*	26	14 (54)	20	31.7	9.0–70.0	35	14	25 (52)	25 (56)
*Listeria*	14	14 (100)	13	26.1	9.3–65.0	38	13	26 (58)	24 (71)
*Propionibacterium*	10	7 (70)	7	49.2	18.0–68.1	13	4	9 (100)	9 (33)
Other§	9	4 (44)	8	41.3	14.8–70.3	14	7	13 (85)	11 (45)
All	618	314 (51)	498	33.6	1.4–71.9	34	336	627 (41)	595 (34)

*n = number identified by mandated reporting. The remainder of isolates were identified by laboratory audit. †No. of isolates for which information on time from inoculation to growth and number of bottles to which samples had been added and number of bottles yielding isolate were available, respectively. Not all laboratories were able to retrieve these data retrospectively for laboratory audits. ‡No *Corynebacterium diphtheriae* or *Bacillus anthracis* organisms were reported. §Other category includes *Bifidobacterium* (2), *Brevibacterium* (2), *Actinomyces* (1), *Aureobacterium* (1), *Erysipelothrix* (1), *Eubacterium* (1), and *Oerskovia* spp. (1).

Among the 498 blood isolates with available incubation period, 171 (34%) isolates grew in <24 hours. Of these, 131 (76%) were reported to CDPH: 97 by telephone (61% reported on date detected and 42% reported outside office hours), 31 by mail, and 2 by unknown reporting method. Overall, 82% of these rapid-growing isolates were either *Bacillus* (52%) or *Clostridium* spp. (30%).

Unreported isolates (n = 304) identified by laboratory audit only grew more slowly (80% incubation period >24 hours versus 54% of reported isolates, p<0.001) and/or presumed contaminants (65% *Corynebacterium* spp.). Nearly all (98%) unreported isolates were from clinical laboratories that had reported other isolates but failed to report all isolates. *Corynebacterium* isolates (all nondiphtheria species, i.e., "diphtheroids") were less likely to be reported than other genera (30% vs. 70%; p<0.001).

*Clostridium* isolates grew significantly more quickly in blood culture than other genera (median incubation 15.3 hours; Table 2) and more frequently in inoculated anaerobic culture bottles (68%) than in aerobic culture bottles (13%). Annualized incidence of clostridial bacteremia was 2.3/100,000 persons, excluding 6 postmortem cultures likely due to agonal bacteremia. The 67 patients were elderly (median age 76 years) and frequently critically ill (22 deaths). Many (56%) had an intraabdominal source identified. Underlying immune-compromise (49%) and malignancy (60%) were common; 24% had neither condition.

**Table 2:** Genus as predictor of incubation time, Connecticut gram-positive rod surveillance, March–December 2003

Genus	No. isolates	Mean incubation (h)	Mean incubation difference* (h)	Standard error	p value*
Clostridium	69	21.1	Ref	Ref	Ref
*Bacillus*	161	28.1	6.99	2.20	0.002
*Listeria*	13	30.7	9.60	4.63	0.038
*Lactobacillus*	20	33.3	12.19	3.89	0.002
*Corynebacterium*	220	43.8	22.62	2.11	<0.001
Other†	15	43.1	21.94	4.36	<0.001

*Mean difference is β-coefficient of univariate linear regression comparing each genus to *Clostridium* spp., the reference group; p value is the p value associated with that β-coefficient. Ref, reference. †Other category includes *Propionibacterium* (7), *Bifidobacterium* (2), *Brevibacterium* (2), *Actinomyces* (1), *Aureobacterium* (1), *Erysipelothrix* (1), and *Oerskovia* spp. (1).

From March to September 2003, an average of 56 staff hours was required per month to receive, respond to, and process reports. For September 2003 specifically, the most recent month assessed, aggregate personnel time was 45 hours (20% outside office hours).

## Conclusions

A major public health preparedness challenge is increasing the sensitivity and timeliness of recognition of individual, potentially sentinel cases of category A bioterrorism agent disease. Each category A agent has unique clinical and diagnostic features: no one system can meet the challenge for all agents. For anthrax, we attempted to shorten the time from occurrence of the earliest specific diagnostic finding, GPR identified by Gram stain of blood or CSF culture, to notification of the public health system. In doing so, we established an around-the-clock GPR laboratory reporting system with <1 full-time staff position. The system has enhanced rapid communication between CDPH and laboratories and provided baseline information on GPR sepsis epidemiology.

The first system objective was earlier detection of anthrax septicemia and meningitis. Additional anthrax cases have not occurred to test this system, and most *Bacillus* isolates are attributable to culture contamination. However, through auditing, we determined that 62% of *Bacillus* isolates identified within 24 hours of inoculation were reported by telephone. Improvement is needed, but, through auditing, the system tracks the timeliness and completeness of reporting and speciation of all *Bacillus* organisms, including, potentially, the next *B*. *anthracis* isolate.

Overcoming laboratory personnel's reticence to report results that are likely spurious culture contaminants has been a challenge of implementing the system. This reticence is reflected by the low reporting rate for *Corynebacterium* spp. (i.e., "diphtheroids") with their unique Gram stain appearance and rare association with pathology. Despite this, our analysis indicates that the system has met its second objective of ensuring around-the-clock laboratory reporting of potential bioterrorism events, given that many GPR reports were made by telephone outside office hours.

The third system objective was to describe baseline GPR septicemia and meningitis epidemiology. Most clinically important isolates were *Clostridium* spp. Like *B*. *anthracis*, *Clostridium* spp. grow rapidly in blood culture and can produce a life-threatening sepsis syndrome. However, during a repeat anthrax attack, the distinct epidemiology of clostridial sepsis could help differentiate clostridial sepsis from inhalational anthrax among persons who are critically ill with a GPR sepsis. *Clostridium* spp. predominately grow in anaerobic culture bottles, and clostridial sepsis usually affects elderly persons with abdominal conditions, malignancy, or immune suppression (*11**,**12*). Notably, recent clostridial sepsis outbreaks involving contaminated tissue transplants and illicit drugs have an epidemiology different from this baseline, in which illness predominately affects persons <50 years of age (*13**–**15*).

An ongoing challenge to this surveillance approach is that no precise clinical algorithm exists for how to readily identify whether a bacterium isolated from blood culture is from culture contamination. This uncertainty complicates the triage of isolates' clinical importance even with physician consultation.

The GPR surveillance system continues with modification. Beginning January 2004, Connecticut laboratories are now required to report by telephone any blood or CSF specimen with growth of GPRs within 32 hours of inoculation. Growth after 32 hours is no longer reportable, to reduce reporting of culture contaminants without significantly sacrificing sensitivity to detect anthrax or clostridial infections. Immediate clinical follow-up is conducted on isolates most likely to be sentinel events: aerobic bottle isolates (possible anthrax event) and anaerobic isolates in patients <50 years of age (unusual *Clostridium* event).

The earliest possible knowledge of an anthrax attack could minimize illness and death by allowing more lead time for intervention. Connecticut has successfully implemented a laboratory-based system that allows for early detection of even a single case of inhalational anthrax.
